# Profiling RE1/REST-mediated histone modifications in the human genome

**DOI:** 10.1186/gb-2009-10-1-r9

**Published:** 2009-01-27

**Authors:** Deyou Zheng, Keji Zhao, Mark F Mehler

**Affiliations:** 1 Institute for Brain Disorders and Neural Regeneration, Department of Neurology, Rose F Kennedy Center for the Study of Intellectual and Developmental Disabilities, Albert Einstein College of Medicine, Morris Park Avenue, Bronx, NY 10461, USA; 2 Department of Genetics and Neuroscience, Albert Einstein College of Medicine, Morris Park Avenue, Bronx, NY 10461, USA; 3 Laboratory of Molecular Immunology, National Heart, Lung and Blood Institute, National Institute of Health, Rockville Pike, Bethesda, MD 20892, USA; 4 Departments of Neuroscience, and Psychiatry and Behavioral Sciences, Einstein Cancer Center, Albert Einstein College of Medicine, Morris Park Avenue, Bronx, NY 10461, USA

## Abstract

**Background:**

The transcriptional repressor REST (RE1 silencing transcription factor, also called NRSF for neuron-restrictive silencing factor) binds to a conserved RE1 motif and represses many neuronal genes in non-neuronal cells. This transcriptional regulation is transacted by several nucleosome-modifying enzymes recruited by REST to RE1 sites, including histone deacetylases (for example, HDAC1/2), demethylases (for example, LSD1), and methyltransferases (for example, G9a).

**Results:**

We have investigated a panel of 38 histone modifications by ChIP-Seq analysis for REST-mediated changes. Our study reveals a systematic decline of histone acetylations modulated by the association of RE1 with REST (RE1/REST). By contrast, alteration of histone methylations is more heterogeneous, with some methylations increased (for example, H3K27me3, and H3K9me2/3) and others decreased (for example, H3K4me, and H3K9me1). Furthermore, the observation of such trends of histone modifications in upregulated genes demonstrates convincingly that these changes are not determined by gene expression but are RE1/REST dependent. The outcomes of REST binding to canonical and non-canonical RE1 sites were nearly identical. Our analyses have also provided the first direct evidence that REST induces context-specific nucleosome repositioning, and furthermore demonstrate that REST-mediated histone modifications correlate with the affinity of RE1 motifs and the abundance of RE1-bound REST molecules.

**Conclusions:**

Our findings indicate that the landscape of REST-mediated chromatin remodeling is dynamic and complex, with novel histone modifying enzymes and mechanisms yet to be elucidated. Our results should provide valuable insights for selecting the most informative histone marks for investigating the mechanisms and the consequences of REST modulated nucleosome remodeling in both neural and non-neural systems.

## Background

The repressor element 1 (RE1) silencing transcription factor (REST; also known as neuron-restrictive silencing factor (NRSF) or X box repressor (XBR)) is the first system-wide transcription repressor implicated in vertebrate neuronal development [1-5]. Since its initial discovery as a repressor binding to RE1 sites in the SCG10 [2], type II sodium channel [5], and synapsin I [6] genes, REST has been shown to repress expression of more than 30 neuronal genes in non-neuronal cells [7]. Its roles have also expanded from the original proposed master regulator of neuronal gene expression [7] to include diverse biological processes and various disease states, including neurodevelopmental and neurodegenerative diseases, stroke, epilepsy, cardiomyopathies, and cancer [8-12]. The profound context-specificity of the functional repertoire of REST and its intricate and evolving regulatory network are further underscored by its dual role as a tumor suppressor and concurrently as an oncogene [11,13,14].

The Kruppel-type zinc finger domain of REST recognizes the RE1 (also known as neuron-restrictive silencer element (NRSE)), a 21 bp DNA element. RE1 nucleotide composition has been characterized extensively and several probabilistic models (that is, position specific frequency matrices (PSFMs)) for the RE1 motifs have been independently developed by several research groups [7,15-18]. An extensive comparison of these models and their relative successes in detecting functional RE1 motifs has so far not been addressed, but the high information content in the 21 bp RE1 motif, due in large part to its long length and high sequence conservation, suggests that high-affinity RE1s can be identified by any of the proposed models. Nevertheless, these models will certainly show differences in recognizing functional but low-affinity RE1s because of the prevalence of non-functional sequences that contain only one or two mismatches to genuine RE1 motifs. Such RE1 mimic sites are especially enriched in repetitive sequences of the human and mouse genomes [16,19,20]; moreover, they have been proposed as a genomic reservoir for the evolution of novel RE1 functional sites [16,19]. For instance, a significant number of human endogenous retroviruses and long interspersed nuclear elements (particularly type 2 (L2)) contain sequences matching RE1 motifs [16]. The presence of RE1 motifs in L2 is very interesting because L2 is an ancient transposon present before the divergence of the human and rodent lineages. Some of these L2 RE1s have been shown to interact with REST *in vitro *[16], although their *in vivo *activities and functional repertoires remain to be defined.

Recently, the association of REST with RE1s *in vivo *has been characterized genome-wide using chromatin immunoprecipitation (ChIP) assays coupled with high-throughput sequencing - ChIP-Seq [19], ChIP-PET [21], or SACO (serial analysis of chromatin occupancy) [20]. In addition to the identification of several thousands of REST bound regions in the human and mouse genomes, these studies have also uncovered a new type of REST binding motif. Unlike many transcription factor binding sites with palindromic sequences, the RE1 motif is not symmetrical and can be divided into two distinct halves, each consisting of a 10 bp sequence. The canonical RE1s (cRE1s) contain a single non-conserved residue between the two halves; the new motifs from genome ChIP assays, however, are not 21 bp long, as the middle insertion varies from 0, or 3-9 bp [16,20]. Not only are these non-canonical RE1s (ncRE1s) able to interact with REST, but they can also mediate gene regulation just like their canonical counterparts [16,20]. Furthermore, some REST bound regions contained only half of the cRE1 motif [19,21], suggesting that local chromatin environment might affect the interaction between RE1 and REST. Nevertheless, the nucleotide composition of the ncRE1s appears highly similar to that of the cRE1s, indicating that the binding of REST is very sequence-specific. No significant differences have as yet been identified in comparing the functional categories of genes with canonical or ncRE1s [19,20].

With recent advances in characterizing the interaction between REST and its cognate DNA (that is, RE1s), our understanding of REST functions has also evolved from the original view of its seminal role in repressing neuronal genes in non-neuronal cells to a more elaborate comprehension of the overall REST regulatory network. The fact that the majority of RE1s are not located in promoters but rather in regions distant (>50 kb) from promoters [16,19,20] suggests that REST functions can be complex, multi-layered, and genome-wide. First of all, REST expression itself is tightly regulated at multiple steps, ranging from transcriptional and post-transcriptional to translational and post-translational processes [11,12,22]. For example, the REST gene is highly expressed in most embryonic and adult non-neuronal cells but at much lower levels in differentiated neurons [22]. This regulation is achieved, in part, through the use of three alternative 5' exons, the production of four protein isoforms, and the presence of multiple regulatory elements in the promoter regions [10], including a retinoic acid receptor element [23]. REST isoforms can interact differently with RE1s and at least one isoform (REST4) has even been implicated in differential nuclear localization, modular function, and gene activation in neurons [24-26]. Interestingly, the inductive role of REST4 is mediated, in part, by the nucleosome remodeling factor BRG1 (see below), which is recruited to the REST complex in the presence of glucocorticoid ligand-dependent transcription [25]. Also, the REST-interacting LIM domain protein (RILP) has been implicated in the traffic of REST isoforms between nucleus and cytoplasm [27]. Moreover, the existence of a ncRE1 in the REST gene suggests a possible autoregulation of REST via a negative feedback loop [19], and the presence of a retinoic acid receptor element in the REST promoter indicates the role of retinoic acid receptor in the repression of the REST gene during neuronal differentiation [23]. Adding yet another layer of complexity to the REST regulatory network is its involvement in regulating many non-coding RNAs [17-20,28]. For example, REST has been shown to regulate the expression of several mouse microRNAs (*mir-9*, *mir-124 *and *mir-132*), all of which promote neuronal differentiation [28]. More intriguingly, a small double-stranded RNA containing RE1 (dsNRSE or RE1 dsRNA) has been identified and shown to interact with REST and modify its function from silencing to activating neuronal genes in adult rat neuronal stem cells [29].

Nevertheless, central to the REST regulatory network is chromatin remodeling mediated by a variety of proteins that interact with REST either directly or indirectly. It is now clear that REST does not act alone; the dynamic and multi-faceted roles of REST are achieved through distinct modular macromolecular complexes recruited by REST. Thus, REST serves as a hub for recruiting multiple chromatin modifying proteins, including multiple histone deacetylases (HDACs) and lysine specific demethylases (LSDs; for example, LSD1) [8,10,30]. These histone modifiers interact either directly with REST or its corepressors, CoREST [31] and mSin3 [32-35]. The histone methyltransferase G9a, the NADH-binding factor CtBP, the methyl-CpG binding protein MeCP2, and the SWI/SNF ATP-dependent nucleosome remodeling factor BRG1 are other currently known factors recruited to the REST complexes for chromatin remodeling [10]. Several histone residues and their modifications have been identified as targets of these REST recruits: H3 and H4 lysine acetylations for HDAC1/2 [32-35], H3K4 methylations for LSD1 [36], H3K9 and H3K27 methylations for G9a [37], and H4K8 acetylations for BRG1 [38,39]. A second lysine demethylase, SMCX, has also been found to interact with REST to facilitate the removal of tri-methyl modifications on H3K4 (H3K4me3) and has specifically been implicated in autism as well as mental retardation [40]. Heterochromatin protein 1 via its association with G9a and methylated H3K9 is also functionally linked to RE1/REST regions [41]. As a result of the recruitment of these diverse chromatin-modifying factors, several histone post-translational modifications implicated in gene activation are removed from the nucleosomes in RE1 regions upon REST binding whereas other modifications associated with gene repression are added. These modifications in turn create a platform for readers (or effectors) of histone code [42] to orchestrate key biological processes for the establishment and maintenance of short- and long-term silencing of genes harboring RE1 motifs. The considerable degrees of interdependence and cooperation between multiple DNA, histone and nucleosome modifying enzymes recruited by REST suggest that more systematic and comprehensive investigations are needed to elevate our understanding of the intricate and nuanced roles of REST in neural development, organogenesis, human disease states and as potential disease biomarkers and novel therapeutic targets.

In this study, we have characterized RE1/REST-dependent chromatin remodeling in terminally differentiated cells, specifically human T cells. With a genome-wide map of REST bound regions and a set of 38 histone modifications (Table 1) mapped across the entire human genome at high-resolution, we have for the first time been able to systematically explore the diversity, magnitude, and potential consequences of chromatin modifications coordinated by REST complexes. We herein demonstrate that binding of REST to RE1 motifs results in nucleosome repositioning accompanied by profound reductions in histone acetylations and declines in selected histone methylations (for example, H3K4me) associated with gene activation, but increases in other methylations (for example, H3K27me3) implicated in gene repression. These patterns of histone modifications were not only detected in promoters with RE1-bound REST, but more intriguingly were also seen in the subset of genes exhibiting upregulated expression. Our analyses have also shown that REST-mediated chromatin remodeling is not restricted to promoter regions and that the interactions of REST with cRE1s and ncRE1s overall have similar epigenetic and functional outcomes. Moreover, our study has defined the correlations among REST occupancy, the strength of RE1 motifs, and the extent of various histone modifications. Our integrated analyses provide critical information for studying the role of REST in mediating different types and degrees of chromatin remodeling, nucleosome dynamics, and gene expression in other cell systems and in various disease states that have been linked to complex and diverse epigenetic lesions.

**Table 1:** REST-mediated changes in histone modifications in RE1 regions

Factor	Promoter cRE1	Non-promoter cRE1	Promoter ncRE1	Non-promoter ncRE1
H2AK5ac	-	-	--	--
H2AK9ac	-	NC	-	-
H2BK120ac	--	-	--	--
H2BK12ac	--	-	--	--
H2BK20ac	--	-	--	--
H2BK5ac	--	-	--	-
H3K14ac	-	NC	-	NC
H3K18ac	--	-	--	--
H3K23ac	NC	NC	-	NC
H3K27ac	--	-	--	-
H3K36ac	--	-	--	--
H3K4ac *	--	-	--	--
H3K9ac	--	NC	--	NC
H4K12ac	-	--	-	--
H4K16ac	-	-	-	-
H4K5ac	--	-	--	--
H4K8ac	--	-	--	--
H4K91ac	--	-	--	--
H2BK5me1	+	-	+	-
H3K27me1	-	-	-	-
H3K27me2	+	+	+	+
H3K27me3	+	+	+	+
H3K36me1	NC	NC	NC	NC
H3K36me3	-	-	-	-
H3K4me1	--	-	-	--
H3K4me2	-	NC	-	-
H3K4me3	-	NC	-	NC
H3K79me1	-	-	--	--
H3K79me2	--	-	--	--
H3K79me3	--	-	--	--
H3K9me1	-	+	-	-
H3K9me2	+	+	+	+
H3K9me3	+	+	+	+
H3R2me1	NC	+	NC	NC
H3R2me2	NC	NC	NC	NC
H4K20me1	NC	NC	NC	-
H4K20me3	NC	NC	NC	NC
H4R3me2	+	NC	NC	NC
H2AZ	--	-	--	-
PolII	--	-	--	--

RE1 regions with bound REST showed increased (plus signs) or decreased (minus signs) histone modifications when compared to RE1 sites without REST occupancy. Modifications without an apparent difference are indicated by 'NC' (for no change), and two minus signs (--) mark a larger magnitude of change than one minus sign (-).

## Results

### Identification of RE1 sites in the human genome

Several groups have independently described their own PSFMs for identifying RE1 motifs [7,16-18,20], but a consensus RE1 PSFM has not emerged. Here, we have applied the method and PSFM developed previously for the program Cistematic [17] to the human genome, and identified 1,333 cRE1 and 2,375 ncRE1 motifs. Of these cRE1s and ncRE1s, 315 (23.6%) and 613 (25.8%), respectively, overlap with repetitive elements, consistent with the known close similarity between RE1 motifs and human endogenous retrovirus or L2 [16]. By intersecting these RE1s with REST bound regions, defined by the ChIP-Seq data from the Jurkat T cell line [19], we found that most of the RE1s embedded within repeats are unlikely to be bound by REST, as 30.2% and 1.1% of those cRE1 and ncRE1 sites, respectively, overlapped REST-enriched regions. In contrast, significantly higher percentages of the non-repeat cRE1 (71.1%) and ncRE1 (11.5%) sequences were found to occupy by REST. These data suggest that: most RE1 sites in repetitive regions are probably inaccessible to REST; and the *bona fide *biochemical motif for ncRE1 is likely more diverse than what was used here, which is essentially the cRE1 PSFM split into two halves. Nevertheless, the number of functional ncRE1s is expected to be much smaller than that of cRE1s based on whole genome ChIP analysis [19].

### Binding of REST in promoter regions is associated with downregulation of gene expression

**Figure 1:**
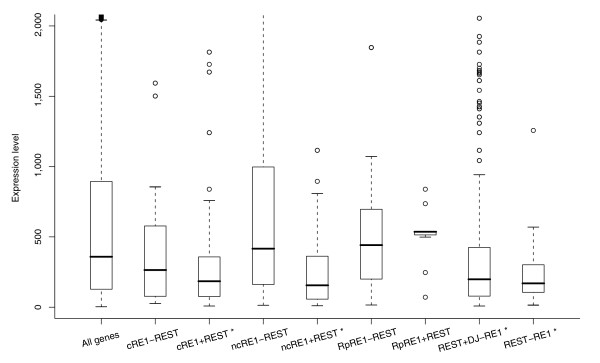
RE1 and REST-mediated gene repression. The expression levels in CD4+ T-cells are shown as boxplots for all human genes (All genes), RE1 genes without REST (cRE1-REST and ncRE1-REST) and with REST (cRE1+REST and ncRE1+REST) in their promoters, and genes with RE1 motifs in the repetitive sequences of their promoters (RpRE1-REST and RpRE1+REST). Conversely, the genes with REST in their promoters are also separated into two groups, one with (REST+DJ-RE1) and the other without (REST-RE1) RE1s annotated in a previous study [19]. An asterisk indicates groups significantly (*P *< 0.001) different from all human genes with respect to their expression scores.

It is generally thought that REST inhibits the expression of neuronal genes in non-neural cells. Based on the microarray data previously published for human CD4+ T-cells [43], the expression of genes with a cRE1 in its promoter was generally lower when compared with the full set of human genes, but such a difference was not obvious for those genes with a ncRE1 (Figure 1). However, the expression was significantly reduced for both cRE1 and ncRE1 genes with REST bound to their promoters. This REST-mediated repression is also seen for genes without a currently annotated RE1 motif. Nevertheless, we should mention that several genes with REST-bound RE1 exhibited expression higher than the median expression level of all genes (for example, *CLK2 *and ZNF638). This is actually consistent with several recent reports showing that REST can sometimes activate gene expression [15,20,25,44], suggesting that the outcome of gene expression upon REST binding can be complex and context dependent even in non-neuronal cells. Since RE1s in repeats appeared not to affect gene expression (Figure 1) and the majority of them did not associate with REST, they were excluded from our subsequent analyses, although their inclusion did not affect our observations and conclusions.

### REST binding promotes nucleosome reorganization surrounding RE1 sites

**Figure 2:**
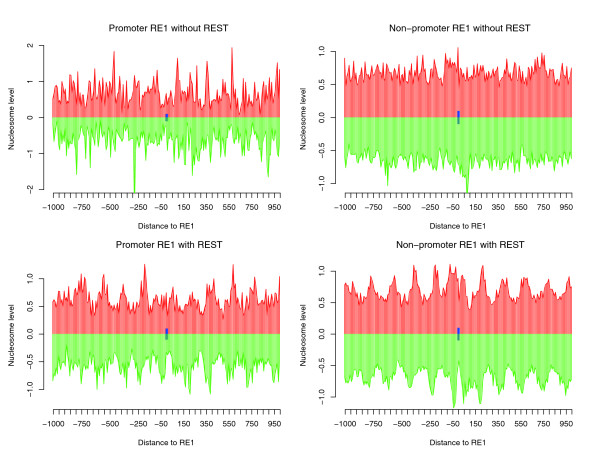
Dynamics of nucleosomes near the promoter and non-promoter cRE1 modulated by REST binding. The y-axis shows the normalized number of sequence tags (in a 10 bp window) from the sense strand (red) and antisense strand (green). The x-axis shows the distance to the center of canonical RE1s (blue box).

We first examined the nucleosome positions in cRE1s using data obtained from high-throughput sequencing of nucleosome ends [45]. The nucleosomes flanking the RE1 sites with bound REST were strongly phased/positioned in the non-promoter regions (Figure 2). At least five phased/positioned nucleosomes on each side of RE1s could be observed. Similar, albeit weaker, nucleosome positioning was observed surrounding the promoter RE1 sites. In contrast, only one positioned nucleosome present directly over the RE1 sites was detected in RE1 regions without REST presence, suggesting that these RE1s may not be accessible to REST. Compared to cRE1s, weaker nucleosome positioning/phasing occurred near ncRE1 sites bound by REST (data not shown).

### REST binding correlates with reduced histone acetylation in promoters

**Figure 3:**
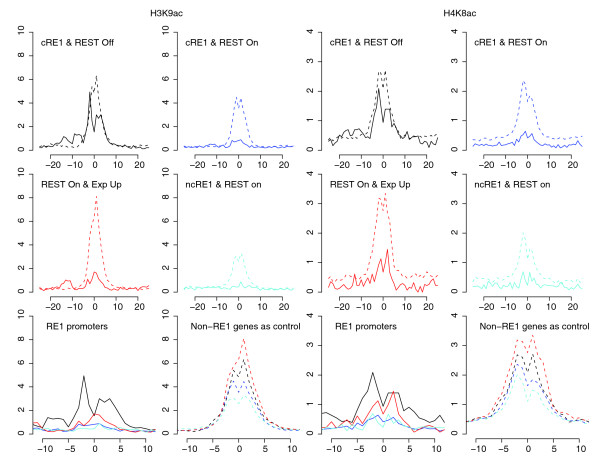
H3K9ac and H4K8ac profiles in RE1 promoters. The profiles of these acetylations were generated and plotted for four groups of genes with different colors (black, blue, red, and cyan), defined by the presences of cRE1, ncRE1, and REST in their promoters. The 'REST On & Exp Up' (red lines) refers to the group of genes with cRE1 and REST but an expression score >300. The profiles of modifications for these RE1 genes are shown with solid lines. For each of the four groups, a control was constructed by randomly selecting (5×) genes with the same expression levels but with neither RE1 nor REST in their promoters (see Materials and methods). The profiles of these controls are shown with dashed lines and colors matching to their targeted group. For the convenience of visual comparison, the zoom-in profiles for the four RE1 groups and their controls are re-drawn in the bottom panels. The color scheme and line style in the bottom panels apply to Figures 5-7. The x-axis shows the distance to transcription start sites with a unit representing 200 bp, and the y-axis shows the normalized counts of ChIP-Seq tags.

Having observed the effect of REST on nucleosome phasing, we next investigated REST's roles on individual histone modifications. As described above, REST regulates gene expression through recruiting multiple modular corepressor complexes. In particular, two of its corepressors, mSin3 and CoREST, can further recruit HDACs (HDAC1/2) [8,10,23]. In order to more fully characterize REST-mediated histone deacetylation, we decided to initially focus on RE1 genes (that is, genes with a RE1 in their promoters) and to examine the profiles of histone acetylation around their transcription start sites (TSSs). In total, 148 human genes had a cRE1, 115 of which also had REST bound to their promoters. A comparison of these 115 cRE1/REST promoters and the remaining 33 cRE1 genes without REST showed clearly that binding of REST to RE1s correlated with dramatic reduction in the acetylation of H3K9 (Figure 3), a known target of HDACs [10,46].

As gene repression is intimately correlated with histone hypoacetylation [47], it is necessary to address to what extent the observed histone deacetylation is merely a reflection of gene repression rather than the direct target of REST complexes. Therefore, we created two sets of genes as our controls. Both control sets consisted of genes with neither an RE1 motif nor REST occupancy in their promoter regions, but one set contained randomly chosen genes whose expression profiles matched that of cRE1/REST genes while the other set exhibited expression as diverse as that of cRE1 genes without REST binding. As such, the difference of a histone modification between these two sets served as a reference for us to determine the change contingent on gene expression but not due specifically to REST occupancy on RE1 sites. As shown here (Figure 3 and figures below), this strategy is highly informative, and after taking into consideration the information in our controls, we concluded that much of the reduction in H3K9ac was in fact a direct consequence of REST binding (Figure 3).

**Figure 4:**
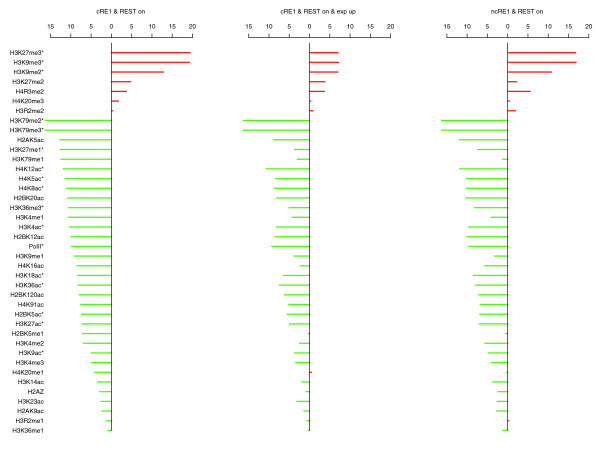
The *P*-values of paired *t*-test for comparing profiles between cRE1 promoters without REST and cRE1 with REST (or ncRE1 with REST, or cRE1 with REST and an expression value > 300). The data for increased and decreased levels of modifications upon REST binding are shown in red and green, respectively. Numbers are -log(10) transformation of *P*-values. An asterisk indicates histone modifications whose *P*-value from the comparison of RE1 genes is <0.0001 and at least ten times smaller than that from contrasting the corresponding control groups.

Further investigation of 17 additional lysine residues (Table 1) in histones H2, H3, and H4 revealed significant REST-mediated deacetylation in the following residues: H4K12, H4K5, H4K8, H3K4, H3K18, H3K36, H2BK5, H3K27, and H3K9 (in order of decreasing significance; Figure 4). As shown in Figure 3, the promoter profiles of H4K8ac and H3K9ac demonstrated clearly that the binding of REST to cRE1 sites correlated with reduced levels of histone acetylation. In both cases, the magnitudes of deacetylation are significantly larger than what were observed in their respective control groups (Figure 3). For some other lysine residues the reduction of their acetylations was prominent and significant, but the change was not always greater than what was observed in their corresponding controls (those not marked with an asterisk in Figure 4). Moreover, reductions of some specific acetylations appeared more contingent on gene repression than others (for example, H3K9ac versus H4K8ac; Figure 3). While the systematic decline of histone acetylations likely results from the actions of HDACs recruited by REST, the decrease of H4K8ac appears to be inconsistent with a previous suggestion that an increase of H4K8ac would facilitate and stabilize the binding of REST to RE1s through the association of REST/CoREST and BRG1 [39], whose bromodomain recognizes acetylated H4K8 (see Discussion).

As previously mentioned, REST binding to a promoter does not always result in gene repression. However, our analyses have revealed that even the upregulated cRE1 genes exhibited REST-dependent deacetylations for most of the lysine residues interrogated (Figures 3 and 4). The REST-mediated histone deacetylations were also analyzed for REST bound ncRE1 genes. The magnitude of the reductions in histone acetylations was largely comparable between REST-bound ncRE1 genes and REST-bound cRE1 genes, in contrast to cRE1 genes without REST (Figures 3 and 4). Therefore, our results demonstrate convincingly that binding of REST to RE1 promoters facilitates significant and broad histone deacetylations.

### REST binding correlates with reductions in histone methylations implicated in gene activation

**Figure 5:**
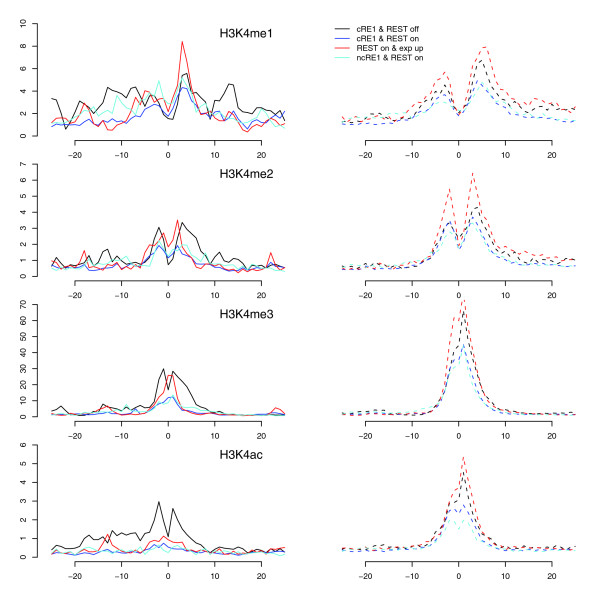
H3K4 profiles in RE1 promoters. The profiles are drawn in the same style as the bottom panels of Figure 3. The y-axis applies to a RE1 group and its control (dashed lines).

The extent of methylations on several lysine residues was also found to be low in the group of cRE1/REST genes. In addition to HDACs, LSD1 and SMCX are two other known histone modifiers recruited by REST to remove H3K4 methylations. Our data reveal that the cRE1/REST promoters had relatively lower amounts of H3K4 methylations than the cRE1 promoters without REST (Figure 5). The magnitude of the difference, however, was smaller than what was seen for H3K4 acetylation, and appeared more prominent for H3K4me2 and H3K4me3 than for H3K4me1 (Figure 5). However, these reductions appeared inextricably linked to gene expression, since the decline in H3K4 methylations was also very noticeable in the genes of our controls, so that the changes in these three methylations became statistically less significant by our measurement, especially for the group of upregulated cRE1 genes (Figures 4 and 5). This observation is consistent with a recent finding that the extent of H3K4me2/3 in several neuronal genes was not affected by the introduction of a dominant negative form of REST into the MPH36 neural stem cell line [44].

**Figure 6:**
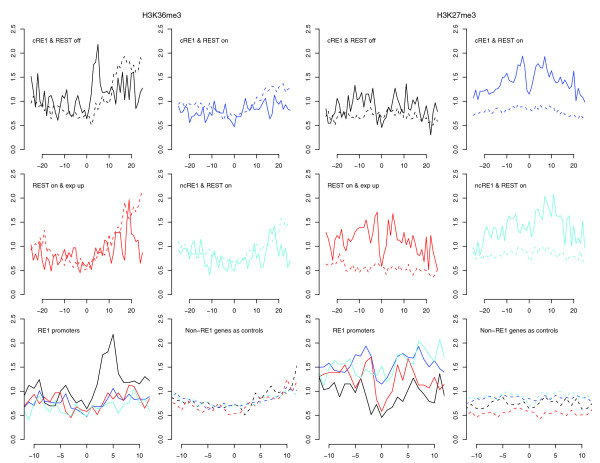
H3K36me3 and H3K27me3 profiles in RE1 promoters, drawn in the same style as Figure 3. The y-axis applies to a RE1 group and its control (dashed lines).

In addition to H3K4 demethylations, REST also reduced the levels of H3K27me1, H3K36me3 (Figure 6), H3K79me3, H3K9me1, H2BK5me1, and H4K20me1; all of these methylation marks are enriched in the promoters of active genes [47,48]. Whereas the enzymes for removing mono- (LSD1), di- (LSD1 and SMCX) and tri-methylation of H3K4me3 (SMCX) are known to interact with REST/CoREST [36,40,49,50] and LSD1 has also been suggested to act on H3K9me [51], our data suggest that additional demethylases could be recruited by REST because LSD1 and SMCX appear unable to remove H3K36me3, H3K79me, and several other methylation marks studied here [36,40,46], although some recently identified JmjC domain-containing histone demethylases exhibit mixed activity profiles for H3K4 and H3K9 methylations [52,53].

### REST binding correlates with enhancement of histone methylations implicated in gene repression

**Figure 7:**
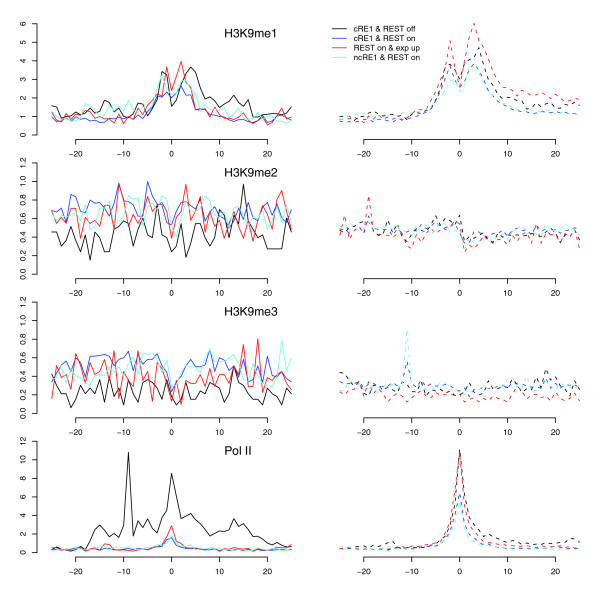
H3K9 and Pol II profiles in RE1 promoters. The profiles are drawn in the same style as the bottom panels of Figure 3. The y-axis applies to a RE1 group and its control (dashed lines).

While no histone residues in REST-bound cRE1 promoters exhibited increased acetylation, several histone residues in these regions displayed high amounts of methylations, including H3K27me2, H3K27me3, H3K9me2, H3K9me3, and H4R3me2 (Figure 4 and 6). These modifications are known to promote general gene repression [47,48], but the surges in H3K27me3, H3K9me2, and H3K9me3 were higher than what were observed in our control gene sets, indicating that these changes are not simply a reflection of gene repression but are directly relevant to REST. In addition, it has been reported that G9a, with a RING finger-like motif that interacts with the carboxy-terminal domain of REST, could increase the methylations in H3K9, predominantly di-methylation in nucleosomes within 2-kb regions of RE1s [41]. Our analyses demonstrate that REST binding increased H3K9 di- and tri-methylations but, surprisingly, reduced H3K9 mono-methylations (Figures 4 and 7). We are not certain whether the relatively uniform distribution of H3K9me2/me3 (that is, no peak was detected) across TSSs could have contributed to this intriguing observation, but we think the phenomenon might be a consequence of competitive interaction between G9a and LSD1, and a conversion of mono- to di- and tri-methylations. Since G9a is not known to methylate H3K27 *in vivo *[41], our data suggest that REST likely interacts with additional histone methytransferase(s), such as polycomb repressive complexes (PRCs).

### REST binding has a similar influence on histone modifications in promoter and non-promoter RE1 sites

We have also characterized the profiles of histone modifications near RE1 sites that are not in promoter regions. Here, the profiles of histone modifications were anchored on the centers of RE1 motifs (rather than TSSs for an obvious reason). Such profiles were separately generated for non-promoter RE1s with and without REST occupancy; for the convenience of comparison, the profiles of histone modifications for promoter RE1s were also re-constructed using the new anchoring system. Comparisons of the resulting profiles demonstrate that, like binding to promoter RE1s discussed above, the association of REST to non-promoter RE1s also resulted in histone deacetylations and selective alterations of a number of heterogeneous histone methylation profiles (Table 1). The outcomes for canonical and ncRE1s were almost identical, though minor and subtle variations existed. It is interesting to note that H3R2me1/2 did not exhibit a change in our analyses, as they are often associated with heterochromatin and generally do not affect gene expression [46] (Table 1). Moreover, not all histone modifications implicated in gene activation (for example, H3K36me1) or repression (H4K20me) displayed a detectable change. Taken together, our data (Table 1) indicate that REST-mediated histone modifications are more prominent at, but not restricted to, RE1s in promoter regions.

### Correlation between RE1 motif strength, REST binding, and histone modifications

As shown in Figures 3, 4, 5, 6, and 7, binding of REST to ncRE1s caused dramatic loss of histone acetylations and several key histone methylations, for example, H3K4me1 (Figure 5), H3K27me1 and H3K36me3 (Figure 6). For most histone modifications, the patterns of change were very similar for cRE1 and ncRE1 genes. However, to our initial surprise, the magnitude of these changes appeared to be larger in ncRE1 than cRE1 sites when REST-bound ncRE1 and cRE1 genes were compared in reference to cRE1 genes without REST. Upon close inspection, we found that the average of our ncRE1 PSFM scores was higher than that of cRE1s (data not shown), suggesting that the degree of REST-mediated histone modifications may be affected by the affinity of a RE1 motif for REST. Such a correlation would also explain the significant correlation of PSFM score with the strength of gene repression regulated by REST [17].

In order to characterize this important observation in detail, we examined all promoters bound by REST and utilized the RE1 motifs and their normalized PSFM scores provided by Johnson *et al*. [19]. Those RE1s are referred to as DJ-RE1 motifs here, which were generated with a lower threshold of PSFM score than what was used in our own RE1 identification process; they therefore represented an expansion of our lists of RE1 sites (and consequently genes). We then computed the correlations between the PSFM scores of the canonical DJ-RE1s in REST-bound promoters and the extent of various histone modifications (using the total number of ChIP-Seq reads within ± 500 bp of DJ-RE1s as a metric). The results clearly demonstrate that the amounts of all histone acetylations were negatively correlated with the strength of RE1 motifs (Table 2). Many of these correlations were quite strong and highly significant, such as those for H4K91ac, H2BK120ac, H3K4ac, and H3K9ac (r < -0.2; Table 2). The PSFM scores of RE1 motifs also appear to be strongly but negatively correlated with several histone methylations, including H3K36me1, H3K4me3, H3K27me1, H3K4me2, H3K79me1, and H3K9me1 (Table 2). For those methylations positively correlated with the RE1 scores, the correlation coefficients were relatively lower, but good correlations existed for H3K9me2, H4K20me3, and H4R3me2. Interestingly, we found that the correlation was positive for H3K9me2 but negative for H3K9me1 (Table 2), suggesting a possible conversion of mono- to di-/tri-methylations. The levels of H2A.Z (r = -0.156) and Pol II (r = -0.132) present in promoters also showed a negative correlation with the strength of RE1 motifs, consistent with RE1's general role in repressing transcription.

**Table 2:** Pearson correlation coefficients between the levels of histone modification and PSFM score, and REST occupancy

Factor	PSFM scores of DJ-cRE1	REST ChIP-Seq reads
H2AK5ac	-0.119	-0.141
H2AK9ac	-0.074	0.138
H2BK120ac	-0.226*	-0.061
H2BK12ac	-0.202*	-0.101
H2BK20ac	-0.220*	-0.055
H2BK5ac	-0.174*	-0.072
H3K14ac	-0.139	-0.054
H3K18ac	-0.177*	-0.102
H3K23ac	-0.13	-0.024
H3K27ac	-0.193*	-0.070
H3K36ac	-0.226*	-0.120
H3K4ac	-0.213*	-0.119
H3K9ac	-0.202*	-0.084
H4K12ac	-0.153	-0.050
H4K16ac	-0.104	-0.053
H4K5ac	-0.136	-0.071
H4K8ac	-0.136	-0.007
H4K91ac	-0.228*	-0.103
H2BK5me1	0.036	0.413*
H3K27me1	-0.121	-0.018
H3K27me2	-0.075	-0.170*
H3K27me3	-0.027	-0.187*
H3K36me1	-0.203*	0.012
H3K36me3	-0.026	-0.108
H3K4me1	-0.059	-0.028
H3K4me2	-0.157	-0.041
H3K4me3	-0.200*	-0.093
H3K79me1	-0.125	-0.135
H3K79me2	-0.096	-0.092
H3K79me3	-0.110	-0.044
H3K9me1	-0.147	0.052
H3K9me2	0.116	0.375*
H3K9me3	0.044	-0.091
H3R2me1	0.023	0.273*
H3R2me2	-0.041	0.355*
H4K20me1	0.081	0.418*
H4K20me3	0.13	0.039
H4R3me2	0.102	0.484*
H2AZ	-0.156	-0.086
PolII	-0.132	0.075
PSFM score	-	0.178*

Significant correlations are marked by an asterisk.

A significant and positive correlation was found between the RE1 PSFM scores and the amount of REST occupancy (r = 0.178), in agreement with previous finding that the fraction of RE1 sites occupied by REST increases with RE1 motif scores [19]. This correlation intriguingly did not lead to a highly parallel relationship between RE1 and REST with respect to their separated correlations with individual histone modifications, though the signs of these correlations were consistent; that is, a negative correlation between a histone modification and RE1s was usually accompanied by a negative correlation between REST and this particular modification (Table 2). But the strengths of the correlations were often different. Moreover, no correlation was found between REST occupancy and many histone modifications that exhibited a strong correlation (r < -0.2) with RE1, such as H3K9ac, H2BK20ac, H2BK120ac, and H3K36me1 (Table 2). Conversely, REST abundance was correlated strongly (r > 0.2) with the levels of H4K20me1, H2BK5me1, H3R2me1, H3R2me2, and H4R3me2, but these histone modifications had no or weak correlations with the RE1 PSFM scores (Table 2). While these observations certainly need to be further characterized, with cross-reactions of immunoprecipitation antibodies being considered, they suggest that the relationship between RE1 motifs and REST occupancy is extremely complex and heterogeneous, and perhaps inextricably linked to the modular nature of REST complexes. For instance, as REST uses its amino- and carboxy-terminal domains to recruit two distinct groups of histone modifying enzymes and some REST protein isoforms are truncated at the carboxyl terminus [10,24-26,54,55], these patterns of correlations could be caused by the presence of more than one REST isoform in T cells (see Discussion for more details).

## Discussion

**Figure 8:**
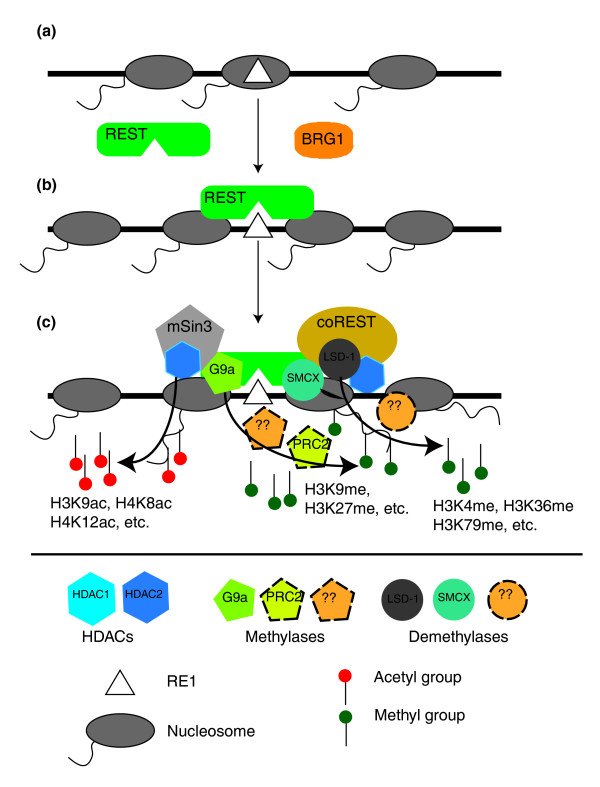
A schematic diagram illustrating the major components involved in REST-mediated local chromatin remodeling and their relationships to our findings. **(a) **RE1 is initially covered by a nucleosome. **(b) **A yet-to-identified cellular mechanism initiates nucleosome repositioning with the assistance of BRG1, resulting in the exposure of the RE1 motif and the subsequent occupation of it by REST. The exact sequential order is not clear to date. **(c) **With the assistance of mSin3 and coREST, the RE1-bound REST complexes then recruit histone deacetylases (HDACs) to promote histone deacetylations, histone methylases (G9a, PRC2) to increase methylations on H3K9 and H3K27, and histone demethylases (LSD1, SMCX) to reduce methylations on H3K4. The presence of PRC2 in REST complexes is unknown but suggested by our analysis, so we have drawn a dashed line around it. Our data also strongly suggest that REST can recruit additional histone methylases and demethylases (represented by question marks) to target other lysine residues of histones, which display RE1/REST-dependent changes in the current study. The enumeration of all the histone modifying enzymes in the REST complexes will enhance our comprehension of how the complicated histone modifications are established; then, more investigations will be needed to decipher how these modifications cross-talk and orchestrate the regulation of RE1 genes.

Since the discovery of REST as a repressor for neuronal genes, many studies have provided significant insights into the cellular and molecular roles of REST in regulating diverse biological processes. What emerges from current literature is a picture of dynamic REST complexes composed of multiple proteins, many of which are involved in differentially establishing and regulating specific profiles of histone modifications or DNA methylation [8,10]. These REST-associated and REST-dependent complexes cooperatively modulate the epigenetic properties in RE1 regions dynamically and help to establish and maintain the cell- and tissue-specific expression patterns of diverse classes of neuronal genes (and non-neuronal genes as well). In this study, we have characterized 38 of 60 known histone modification sites [46,56], and provided a broad overview of how REST macromolecular complexes modulate histone modifications in human T cells. The results of our study can be schematically summarized (Figure 8) in spite of the complexity. Many of our observed changes have been reported previously and, furthermore, the corresponding enzymes have been identified (Figure 8) [10]; thus, strong experimental evidence exists for some of our results, but a significant subset of these histone modifications, particularly those on H2 and H4, are now characterized for the first time in our study. Moreover, our genome-wide analyses have identified some REST-mediated histone modifications (for example, H4K8ac, H3K9me) that extend previous findings based on studying a limited number of neuronal genes to novel observations concerning their putative regulatory roles.

### Promoter and non-promoter RE1s exhibit similar affinity for REST and comparable profiles of REST-mediated histone modifications

Most of our discussions have focused on the RE1/REST interaction in promoter regions of protein coding genes. This is primarily due to the fact that the relationship between histone modifications and gene expression is much better documented for promoters (or near TSSs) than for any other regions [46,47]. The enrichments of many well-characterized histone modifications in promoters [48,56,57] certainly warrant our choice of promoter RE1/REST as the focal point of our report. Nevertheless, our comparisons of histone modifications in promoter and non-promoter RE1 regions produced very similar results in terms of the influence of REST on local histone modifications (Table 1), which strongly suggests that REST coordinated histone modifications are not solely promoter-dependent. This is an important general observation considering that only about 10% of RE1s are located near TSSs, whereas most RE1s are >50 kb away from protein coding genes. Overall, binding of REST to these non-promoter RE1s (or remote RE1s) resulted in similar perturbations of histone modifications as REST binding to promoter RE1s. However, we cannot exclude the possibility that such changes when examined in much greater detail may exhibit more subtle and functional differences. For example, RE1/REST near enhancers may be associated with a distinct pattern of histone modifications. Our study represents the beginning of a much more exhaustive inquiry regarding REST-mediated chromatin remodeling, and in the future we plan to address many of these seminal issues by designing experiments to separately interrogate the differential profiles of chromatin remodeling coordinated by REST but nevertheless occurring within distinct genomic, molecular and cellular contexts.

We did not characterize ncRE1s without REST occupancy in this study. Compared to the high percentage of cRE1s with bound REST, relatively few (11.5%) of our annotated ncRE1s were occupied by REST. We believe this is largely due to the current technical limitation of recognizing genuine ncRE1s in the human genome. Nevertheless, it will be essential to determine whether macromolecular complexes recruited by RE1/REST are substantially different from those recruited by ncRE1/REST, though our data as well as previous studies [19,20] have not sufficiently addressed the existence of such a scenario.

### REST-associated nucleosome reorganization and histone modifications

With integrated high-resolution data, we have begun to illustrate the complex genome-wide landscape of chromatin remodeling coordinated by a single transcription factor. Our results indicate that the majority of RE1 sites are accessible and bound to REST in differentiated T cells. Although we do not know what triggers the association of REST with a particular RE1, our analyses have shown that REST binding induces nucleosome repositioning, profound histone deacetylations, removal of histone methylation marks highly implicated in gene activation, and the addition of selective methylations involved in gene repression (Figures 4 and 8). It needs to be mentioned that cRE1 genes without bound REST also exhibited similar properties but to a much smaller extent compared to genes without RE1s and evidence of REST binding (Figures 3, 4, 5, 6, and 7). This is likely due to the use of a threshold for identifying regions enriched with REST; that is, some REST-free regions might actually be occupied by a small number of REST molecules.

It is important to emphasize that our data provide only a global and static view of the consequence of REST binding, but REST-mediated chromatin remodeling is a highly regulated, cooperative, and sequential process orchestrated by a large number of histone remodeling proteins, with some modifications occurring after and dependent upon earlier modifications [10]. For instance, removal of the acetyl group on H3K9 stimulates LSD1 activity, which subsequently removes methyl groups from H3K4 [49]. Intriguingly, it was previously reported that increased H4K8ac could potentially facilitate the recruitment of REST to RE1 regions. The phase/position of nucleosomes facilitated by REST in RE1 regions (Figure 2) is consistent with this hypothesis as BRG1, one of the ATPases of the SWI/SNF complex with a bromodomain that can recognize H4K8ac, can help reposition nucleosomes with respect to DNA [39]. Our data nevertheless suggest that H4K8ac might be relevant only in the initial recruitment of REST, as it was found to decrease upon REST binding (Figure 3). After the interaction of REST and RE1s is established, however, the subsequent recruitment of HDACs can presumably lead to H4K8 deacetylation.

Our results suggest that histone modification enzymes other than HDACs, LSD1, SMCX, and G9a may also interact with REST and, thus, there are potentially additional and essential components of REST co-repressors (Figure 8). Specifically, our analyses found that REST binding changed the level of several histone methylations that are not known targets of currently identified REST co-repressors. The most noticeable such histone marks are H3K27me2/me3, which were increased upon REST binding, and H3K27me1 and H3K36me3, which were conversely decreased by the presence of REST complexes (Figure 4). Of course, it is possible that these histone methylations are *in vivo *targets of LSD1, SMCX, or G9a, but such catalytic relationships have not yet been elucidated. However, a more likely alternative scenario is that previously unrecognized or never characterized histone modification enzymes are present within distinct REST macromolecular complexes, with PRC2 being a primary candidate as it catalyzes H3K27 tri-methylation (Figure 8) [46]. Moreover, our evolving comprehension of the multiple roles played by individual histone modifications suggests that these REST-associated chromatin remodeling events need to be examined within the broader context of the fine-tuning of local transcriptional control as well as more genome-wide effects on heterochromatin dynamics, boundary elements, and gene networks. Furthermore, it will be highly interesting to study how the RE1/REST sites are enriched for LSD1, SMCX, G9a, HDACs, or other histone modifying enzymes when genome-wide ChIP-Seq data for these factors become available in the future. This is also essential for further unraveling the REST function in detail because it has been shown that the composition of the REST complexes in different RE1 genes could be different [44].

### Histone deacetylases, methyltransferases, and demethylases might have subtle and distinctive roles in transacting REST functions

There is no doubt that binding to RE1s and the subsequent recruitment of histone modifying enzymes are two activities central to the REST regulatory network. These two primary roles of REST are interdependent as reflected by the correlations among RE1 motif score, REST occupancy, and levels of various histone modifications (Table 2). However, the relationships appear much more complex and nuanced as multiple proteins are involved in a sequential and interdependent manner. As a result, we have found that most correlations exist but are not particularly robust as judged by visual inspection or statistical measurement. In particular, we did not detect a significant functional interrelationship between RE1 affinity and REST abundance with respect to their correlations with the degree of individual histone modifications, despite the observation that the correlations occurred in a parallel mode (Table 2). The biochemical specificity and sensitivity of some antibodies in ChIP-Seq might have contributed to this lack of parallel correlations. However, we do not think that such a lack of a strong parallel relationship is caused by the fact that our REST binding data and histone modification data came from different lines of T cells. Instead, we believe that the weak interrelationship reflects the complex amalgam of REST functional roles and dynamic and more global molecular processes. Fundamentally, histone modification is a highly regulated process not solely dependent on REST interactions. It is also known that REST utilizes its amino- and carboxy-terminal domains to recruit at least two distinct groups of histone modifying enzymes. Although it is still largely undefined how these two domains and their associated co-repressors and complementary co-modulatory complexes promote histone and higher-order chromatin crosstalk, REST isoforms with altered or truncated carboxy-terminal domains have been detected in neuronal cells [26,54]. These different REST isoforms must possess a spectrum of different activity profiles associated with their binding of RE1s and the recruiting of selective histone modifying enzymes. In fact, one truncated isoform, REST4, has been found to have a lower affinity for DNA and to activate the expression of neuronal genes by antagonizing the normal function of full-length REST [24-26,55]. Although these REST isoforms have not been reported in T cells, their presence would certainly help to explain at least a subset of our observations regarding the intricate functional interrelationship between RE1 binding affinity and REST occupancy, since our current analyses and the underlying datasets cannot distinguish between different REST isoforms.

We suggest that the amino- and carboxy-terminal domains of REST and their associated histone modifying enzymes might have very distinct and subtle roles in the overall scheme of the REST regulatory network. The HDACs recruited by REST are less selective in their targeted residues, as manifested by the significant reductions in broad histone acetylations observed in our analyses; the histone demethylases and methytransferases recruited by REST are much more discriminative in their molecular targets [10,46,56], as exemplified by the diverse and complex changes in specific histone methylations correlated with REST binding. As a result, HDACs appear to induce a broad repression of RE1/REST genes, whereas the histone demethylases and methyltransferases can cooperate and dynamically alter the profiles of methylations on individual nucleosomes in a more selective, context-specific and nuanced manner, and thus create an elaborate platform for histone code readers [42]. As a result, the interaction of histone methylases and methyltransferases has the potential to fine-tune the expression levels and functions of individual RE1 genes and to integrate gene networks in response to distinct developmental, environmental and interceptive cues and imperatives. Furthermore, the distinct roles of REST-mediated histone acetylation and methylation could be important for multiple developmental processes, as histone methylation has been considered to be more stable than acetylation. In the future, we plan to address our hypothesis with a double immunoprecipitation ChIP-Seq to define how REST and a histone modification are correlated at the molecular level.

### Selection of a subset of histone marks for studying REST-mediated chromatin remodeling

Our results (Figure 4, Table 1) indicate that it may be possible and instructive to use a subset of histone marks to capture the dynamic range of epigenetic modifications orchestrated by REST. Although a genome-wide high-resolution map of histone modifications can be readily obtained with the next generation high-throughput sequencing technology, it is unlikely that we will be able to examine every possible post-translational modification of histones in every cell, particularly in a dynamic fashion required to fully elucidate the functional significance of the integrated higher-order chromatin code and the associated spectrum of epigenetic modifications in the foreseeable future. Therefore, we propose a subset of representative and instructive histone marks that can be used to investigate the overall patterns associated with REST-mediated chromatin remodeling. Based on our results (Table 1, Figure 4) and the observation that many modifications are highly correlated with respect to their patterns of alterations by REST in cRE1 promoters (data not shown), the primary candidates for further study are H3K4ac, H3K9ac, H4K8ac, H3K9me1, H3R2me1, H3K27me3, H3K36me3, and H4R3me2. We believe that a survey of these histone marks will provide considerable insight into the histone modification platforms orchestrated by RE1/REST interaction in cells (or tissues) and genes of interests.

Furthermore, we think that studying histone modifications in a wider variety of cells will be essential for expanding our knowledge of REST functions and will likely be more fruitful than investigating a wider spectrum of histone modifications in a more limited range of cell types over time or in response to specific activation or stressor states. In particular, it will be highly valuable to study whether the complicated and heterogeneous profiles of histone modifications, defined here for RE1/REST in T cells, are specific in non-neuronal cells, and if not, how the patterns evolve in neuronal cells. It is easy to envisage that such a sophisticated and modulated epigenomic remodeling program can play a significant role in neuron differentiation and maturation. We also believe that studies of additional classes of REST interacting factors, such as multifunctional heterochromatin binding proteins (for example, Heterochromatin protein 1, which interacts with G9a [41]), DNA methylation effectors (for example, MeCP2, which recognizes methyl-DNA [58]) and specific subclasses of short and longer non-coding RNAs that may promote sequence-specific chromatin-modifications [59,60], will provide additional mechanistic insights required for an overall understanding of REST-mediated chromatin remodeling.

### REST-mediated histone modifications can be associated with enhancement of gene expression in T cells

The focus of this study has been to elucidate the local and more global influences of REST on histone modifications. As histone modifications, especially those on H3, are intrinsically linked to gene expression [46,47], we have constructed and studied gene control groups to computationally 'uncouple' this linkage in order to determine accurately the changes of histone modifications that depend on REST binding to RE1 sites. Nonetheless, the predominant outcome of REST binding is overall gene repression in T cells (Figure 1), in accordance with the originally proposed role of REST as a transcriptional repressor to silence neuronal genes in non-neuronal cell lineages. At the level of individual genes this is surely more complex and dynamic as we have only examined promoters and one particular aspect of the REST regulatory network - histone modifications - whereas the expression of most genes is regulated at multiple levels and by several interrelated epigenetic mechanisms and both local and global genomic modulatory processes.

We have examined a small group of genes with cRE1 and REST in their promoters that were nonetheless highly expressed in human T cells. These genes, such as *CLK2*, *DPH2 *and *RAB37*, seemingly are not specifically related to neuronal or T-cell development. The histone modification data in the promoters of these genes are very valuable as they have helped to demonstrate that REST binding is the cause of reduced levels of histone acetylations and is not entirely contingent on gene expression, and that the influence of REST on methylations was much more complex than expected (Figure 4). For example, H3K4ac was lower in RE1 genes with REST binding regardless of high or low levels of gene expression, but the degree of H3K4 methylations (especially H3K4me1) was noticeably higher only in the group of cRE1/REST genes with up-regulated expression (red line in Figure 5). Several other methylations whose magnitude of change was relatively more contingent on expression level also exhibited such a pattern (Figure 4), supporting our proposed role of methylations in fine-tuning the expression of RE1/REST genes. In particular, compared to cRE1 genes without REST, H4K20me1 was decreased upon REST binding but slightly increased in upregulated cRE1 genes with REST (Figure 4). The effects of the H4K20me1 histone mark are known to be complex and context-specific, including roles in active transcription, heterochromatin formation, and DNA repair [46], as well as potentially serving as a binding platform for the bifunctional JMJD2A H3K9me2/3 and H3K36me2/3 histone demethylase [61]. The level of Pol II in the promoters of this group of genes is also quite intricate as it is much lower than that of REST-free cRE1 genes (Figure 7), suggesting that transcription initiation might not be the key factor responsible for the increased numbers of transcripts for these genes. Although we cannot exclude the possibility that the REST co-repressors associated with these genes might have a distinct molecular configuration, we found that both the RE1 motif score and the number of REST ChIP reads in these upregulated genes were not statistically different from their corresponding values for downregulated cRE1 genes with bound REST (*p*-value = 0.13 and 0.35, respectively). These observations suggest that either additional pathways unrelated to REST are involved in regulating the histone modifications (and consequently expression) of these cRE1 genes, or other component(s) associated with REST must exist to overcome the demethylation activities of REST-associated LSD1 and SMCX. Non-coding RNAs similar to the dsNRSE in rat neuronal stem cells [29] certainly would be excellent and unique candidates for the latter, since it has been shown that double-stranded RNA in promoter regions can modulate histone modifications [62].

## Conclusions

We have integrated multiple sets of genomic data obtained from motif prediction, gene expression, and ChIP-Seq to characterize in details the complex landscape of nucleosome modifications mediated by RE1/REST interactions. Our study reveals that the binding of REST to RE1 induces dramatic context-dependent chromatin remolding, including nucleosome repositioning/phasing, systematic decline of local histone acetylations and some key histone modifications but increase of a different set of important histone modifications. Our findings show convincingly that REST-mediated chromatin remodeling is extremely dynamic and complex with novel histone modifying enzymes to be identified. Our work provides valuable information for appreciating the complexity of the REST regulatory network, and for further decoding the roles of REST and its corepressors in stem cells, and neuronal and non-neuronal lineage cells.

## Materials and methods

### Identification of RE1 sites in the human genome

The occurrences of the DNA motifs (RE1 sites) recognized by REST were identified using the PSFM from the software package Cistematic [17]. The PSFM was derived from a large set of known instances of REST binding sequences and a set of known negative cases. An efficient motif scanning algorithm was implemented and a conserved threshold of 84% of the best possible score [17] was used to select RE1 sites. Whereas RE1 sites of 21 bp were called cRE1s (cRE1s), ncRE1s (ncRE1s) refer to the RE1s with their left and right half sites (10 bp each) separated by 0 or 3-9 nucleotides. The binding of REST to ncRE1s was discovered recently by genome-wide REST ChIP analyses; the ncRE1 motif has been found to be highly similar to that of cRE1s, except for the non-conserved distance between their two half sites. Therefore, we used the same PSFM for cRE1s and ncRE1s but allowed various nucleotide insertions in ncRE1s. The program RepeatMasker was used to identify repetitive regions in the human genome; then RE1 sites fully embedded in repeats were designated as repeat RE1s. We further segregated RE1s into promoter RE1s and non-promoter RE1s based on their locations with respect to the promoters (-5 kb to +1 kb from the TSS) of known genes. Accordingly, the genes with RE1 or REST in their promoters were then termed RE1 genes or REST genes. The transcription levels of known human genes in CD4+ T cells were obtained from a previous microarray analysis [43]. The data were processed using the Affymetrix software MAS 5.0 (MAS5) and low and not expressed genes had an expression score <200.

### Positioning of nucleosomes in relation to RE1 sites

The positioning of nucleosomes near RE1s was characterized with the genome-wide map of nucleosome positions in resting CD4+ T cells constructed by direct high-throughput sequencing of nucleosome ends [45]. The density of nucleosomes was profiled by totaling the reads mapped to a 10 bp window sliding from -1 kb to +1 kb from the center of cRE1s. The reads aligned to sense and antisense strands were treated separately.

### ChIP-Seq data for REST-bound regions and histone modifications

The human genomic regions bound with REST were obtained from a ChIP-Seq assay using a monoclonal antibody against REST in Jurkat T cells [19]. The REST data included a list of genomic regions with numbers of mapped ChIP-Seq reads. The authors also provided the locations of RE1s (with their PSFM scores) within or adjacent to each of these REST bound regions. These RE1s were called DJ-RE1s as they were generated with a motif score threshold lower than what was used in the current study. This is feasible because the identification of DJ-RE1s was applied only to sequences near genomic regions with REST binding; otherwise, this threshold would result in a great number of false positive RE1s.

The genome-wide data for histone modifications have been described in two previous studies, one targeted at histone acetylations [57] and the other focused on histone methylations (plus H2A.Z and RNA polymerases II) [48]. The specificities of individual antibodies have been described [48,57]. The data are lists of genomic coordinates for individual ChIP-Seq reads that could be mapped to the human genome unambiguously.

### Generation and comparison of aggregated profiles of histone modifications

RE1 sites binding REST were inferred computationally by intersecting the predicted RE1 sites with the REST-bound regions. To construct an aggregated profile of a histone modification for RE1 promoters (or RE1 sites), we summed the ChIP-Seq reads in a window of 200 bp moving from -5 kb to +5 kb of TSSs (or the center of RE1s where applicable). This profile was then normalized by sample size (for example, number of TSSs) to generate average histone modifications spanning TSSs (or RE1s) for subsequent direct comparisons. Moreover, in the comparisons of profiles with and without REST in their promoters, a control group was a set of genes whose expression scores in CD4+ T cells matched to those of genes under investigation. For example, to compare the profiles of cRE1 genes with REST (group A) and without REST (group B), five genes without a RE1 and REST were selected randomly from the pool of all human genes for every gene in group A on the condition that these six genes would have the same expression score. Application of this approach to group A thus yielded a control group A', likewise B' for group B. Paired *t*-test was then used to quantify statistically the difference between groups A and B, and the corresponding *P*-value is shown in Figure 4. In this study, the difference between groups A and B would not be considered significant unless the *P*-value was <0.0001 and at least ten times smaller than the corresponding *P*-value from the comparison of groups A' and B'. The goal was to computationally uncouple the change (of histone modifications) directly modulated by REST from that intimately correlated with gene repression. As shown in Figures 3, 4, 5, 6, and 7, this strategy was both effective and highly informative. The profiles anchored on the center of RE1s were visually compared for determining the outcome of REST binding to promoter and non-promoter RE1s.

### Correlation of RE1 PSFM score, REST occupancy and histone modifications

We used the DJ-RE1s for studying the correlation between the strength of RE1s and the degree of histone modifications, because these RE1 had a bigger range of PSFM scores than those RE1s identified in current work. Moreover, these RE1s were derived from genomic regions known to bind REST *in vivo *[19] and thus should have very low false positive sites. The DJ-RE1s were also used to characterize the correlation between RE1 motif score and REST occupancy, and that between REST binding and histone modifications. The metric for REST occupancy was the number of ChIP-Seq reads obtained from the previous study [19], and the metric for a histone modification (or H2A.Z and PolII) is defined here as the number of ChIP-Seq reads within 500 bp of RE1s. An alternative window size of ± 2 kb yielded similar results.

## Abbreviations

ChIP: chromatin immunoprecipitation; ChIP-Seq: cChIP and high-throughput sequencing; cRE1: canonical RE1; HDAC: histone deacetylase; L2: type 2 long interspersed nuclear element; LSD: lysine specific demethylase; ncRE1: non-canonical RE1; NRSE: neuron-restrictive silencer element; PRC: polycomb repressive complex; PSFM: position specific frequency matrix; RE1: repressor element 1; REST: RE1 silencing transcription factor; TSS: transcription start site.

## Authors' contributions

DZ and MM conceived of the study. KZ's group produced all the ChIP-Seq data for histone modifications and nucleosome positioning. DZ designed the experiments and carried out the analyses. DZ interpreted the results with help from KJ. DZ, KJ and MM wrote the paper together. All authors read and approved the final manuscript.
